# Unusual case of delayed congenital diaphragmatic hernia in Loeys-Dietz syndrome: a case report

**DOI:** 10.1093/jscr/rjaa604

**Published:** 2021-01-31

**Authors:** Gilberto O Lobaton, Y Julia Chen, Eric Jelin, Alejandro V Garcia

**Affiliations:** Department of Pediatric Surgery, Johns Hopkins University School of Medicine, Baltimore MD, USA; Department of Pediatric Surgery, Johns Hopkins University School of Medicine, Baltimore MD, USA; Department of Pediatric Surgery, Johns Hopkins University School of Medicine, Baltimore MD, USA; Department of Pediatric Surgery, Johns Hopkins University School of Medicine, Baltimore MD, USA

## Abstract

Congenital diaphragmatic hernias rarely present after 2 months of age and are typically diagnosed in the perinatal period. Moderate to severe diaphragmatic hernias present with respiratory symptoms, while late-onset hernias have a more varied presentation, depending on the age and content of the hernia. Very rarely, such hernias are found on incidental imaging, in which surgical repair is frequently recommended. A young girl with Loeys-Dietz syndrome and prior abdominal surgeries presents with 1-year history of increasingly severe, intermittent, abdominal and left shoulder pain. Prior imaging incidentally revealed a left diaphragmatic hernia with omentum protruding into the thoracic cavity. This was managed expectantly due to her other medical and surgical issues. Serial imaging revealed that the herniated omentum was increasing in size and symptoms began to develop. An uncomplicated primary thoracoscopic repair was performed. We report the first case of a congenital diaphragmatic hernia in a patient with Loeys-Dietz syndrome.

## INTRODUCTION

Congenital diaphragmatic hernias (CDHs) arise from a malformation of the pleuroperitoneal membrane during embryogenesis and are usually diagnosed by fetal ultrasound in over 73% of cases [1–3]. If not diagnosed antenatally, CDH is often diagnosed in the early neonatal period as patients present with varying degrees of pulmonary hypoplasia and respiratory symptoms [2, 4]. Very rarely, CDH presents beyond the neonatal period, where the reported incidence is between 2.6 and 45.5% [5, 6]. Late presentation can prove to be a diagnostic challenge as symptoms are subtle and can present with pulmonary, gastrointestinal or mixed symptoms. Even more rarely, hernias may be asymptomatic and only identified on imaging for another condition [1, 3, 4]. We report a case of an incidentally identified late-onset diaphragmatic hernia, in a patient with Loeys-Dietz syndrome, corrected with a thoracoscopic repair.

## CASE REPORT

A young girl presented to the emergency department at our children’s center with 1 year of increasingly severe, intermittent, abdominal and left shoulder pain. Medical history included a diagnosis of Loeys-Dietz syndrome at the age of 5 months, complicated by aortic enlargement. Detailed abdominal surgical history included Ladd’s procedure for malrotation, Nissen fundoplication and gastrostomy tube placement at 5 weeks of age, exploratory laparotomy at 3 months of age due to spontaneous intraperitoneal hemorrhage of a wandering spleen, incisional and bilateral inguinal hernia repairs at 9 months of age and a gastro-cutaneous fistula repair at 5 years of age.

At age 5, a magnetic resonance imaging of the thoracolumbar spine was obtained in the setting of scoliosis, in which a small incidental diaphragmatic hernia was diagnosed on the left, posterolateral aspect of the diaphragm. The contents of the hernia initially appeared to be retroperitoneal fat, and the patient was asymptomatic. Given her other medical and surgical issues, the hernia was managed expectantly. Surveillance imaging for her aortic enlargement at ages 10 and 11 revealed a 5 cm × 3 cm herniation of retroperitoneal fat into the left hemithorax.

**Figure 1:**
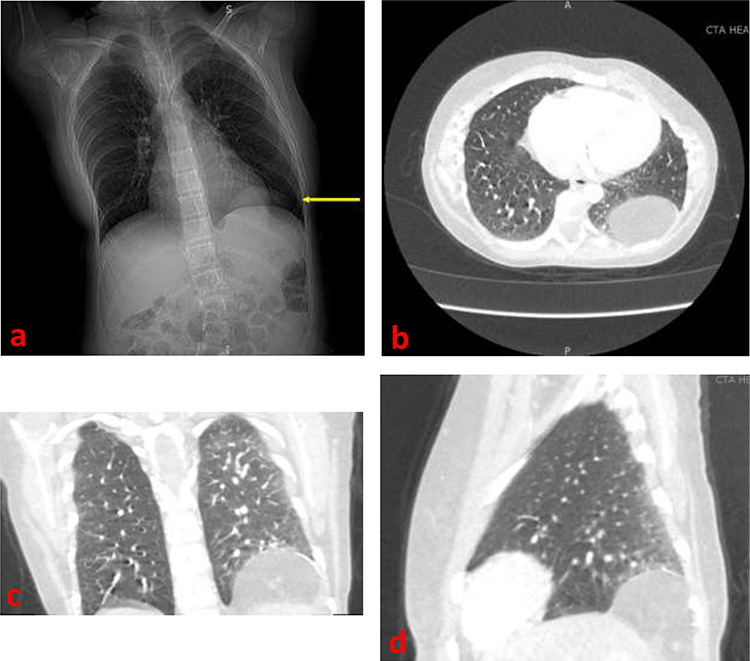
(**a**) Frontal chest radiograph demonstrating a left diaphragmatic hernia (arrow) (**b**) Axial contrast-enhanced CT image demonstrating a left diaphragmatic hernia in the left hemithorax (**c**) Frontal contrast-enhanced CT image demonstrating a left diaphragmatic hernia in the left hemithorax (**d**) Sagittal contrast-enhanced CT image demonstrating a left diaphragmatic hernia.

In the setting of the patient’s new symptoms and her history, a chest radiograph (Fig. 1a) and computed tomography (CT) scan (Fig. 1b–d) were ordered to fully characterize the state of the diaphragmatic hernia. Imaging revealed a 6 × 6 cm protrusion of retroperitoneal fat into the left posterior hemithorax. Given the increased growth in size of the defect that was becoming symptomatic, we performed a video-assisted thoracoscopic repair of the diaphragmatic hernia.

**Figure 2:**
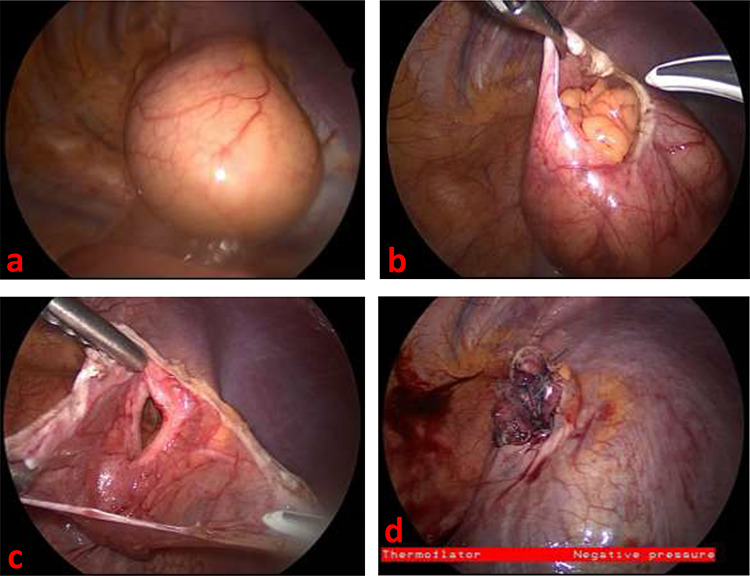
(**a**) Thoracoscopic view of left diaphragmatic hernia upon entry into thorax (**b**) Thoracoscopic view of incarcerated omentum underneath the hernia sac (**c**) Thoracoscopic view of diaphragmatic defect, measuring about 4 cm long, with surrounding hernia sac (**d**) Thoracoscopic view of repaired diaphragmatic defect.

During repair, a large hernia was immediately apparent, protruding from the posterolateral aspect of the diaphragm, consistent with a Bochdalek hernia (Fig. 2a). The hernia sac was opened sharply, and contents appeared to be incarcerated omentum (Fig. 2b). This was reduced into the abdomen, and the hernia sac was excised. The defect measured about 4 cm and was repaired using polyester sutures and pledgets (Fig. 2c and d). Postoperative course was uncomplicated, and the patient was discharged on postoperative Day 4 due to pain control. At follow-up outpatient visit 2 weeks later, she reported complete resolution of her pre-surgery symptoms.

## DISCUSSION

CDH is a potentially fatal defect that generally presents perinatally and is repaired within the first month of life [1, 3, 4]. Very rarely, they can present later, as in our case. In a 10-year study by The Congenital Diaphragmatic Hernia Study Group, proportion of late-onset CDH was 2.6% [6]. Although anatomically the defect is similar, in late-presenting CDH there are extensive differences in symptoms, workup and ultimately management [6].

In our case, frequent surveillance imaging required by our patient’s underlying genetic disorder offered assistance in diagnosing the etiology of her vague but chronic pain. Such an incidental finding is reported to occur in as little as 7 in 10 000 patients [7]. Given the patient’s presenting symptoms of abdominal and radicular left shoulder pain, her known left CDH as the source of symptoms was most likely.

In addition, our patient was without a spleen since early life. In some cases, the spleen may be well positioned to occlude a small Bochdalek hernia [8], however the lack of spleen may have allowed omental fat to begin accumulating in the patient’s thorax over time with episodes of increased intrabdominal pressure. Another potential etiology was an iatrogenically induced diaphragmatic hernia from one of her several abdominal surgeries. However, given the wandering spleen and report of location in the pelvis, coupled with the hernia sac found intraoperatively, it is unlikely that the source of her CDH was iatrogenic.

Our patient also had Loeys-Dietz, a clinical syndrome included within the Marfan spectrum known to cause multiple developmental anomalies including aortic and arterial aneurysms, hypertelorism, bifid uvula/cleft palate, arterial tortuosity, craniosynostosis, brain anomalies, mental retardation and spine deformities [9]. The affected gene in Loeys-Dietz causes perturbations in TGF-beta signaling, a family of cytokines that has vast implications on cellular proliferation, migration and death [9]. There are a wide array of clinical phenotypes associated with TGF-beta signaling disarray, although to our knowledge, there have been no reported cases of Loeys-Dietz and diaphragmatic hernias. It is plausible that this genetic defect may have played a role in the development of the pleuroperitoneal membrane *in utero*, resulting in a diaphragmatic hernia for our patient.

Late-onset CDH can be repaired via thoracic or abdominal approaches. While the abdominal approach is more traditional, there has been increased evidence to suggest that thoracic approach can be just as effective [10]. Our decision to approach this repair through the thorax involved her history of extensive abdominal surgery to avoid the morbidity of potentially prolonged lysis of adhesions and abdominal retroperitoneal exposure. Given the imaging findings that confirmed only the presence of omentum in the hernia sac, the morbidity of an abdominal exploration could be avoided in her case [10].

In summary, we report a rare case of incidental late-onset CDH in an adolescent patient with Loeys-Dietz syndrome and extensive history of abdominal surgery, repaired thoracoscopically. The genetic mechanisms underlying Loeys-Dietz may be associated with the formation of a diaphragmatic hernia, and, to our knowledge, this is the first case report of a CDH in a Loeys-Dietz patient.

## CONFLICT OF INTEREST STATEMENT

None declared.
